# Oil of Turpentine in the Treatment of Yellow Fever

**Published:** 1853-10

**Authors:** 


### RECORD OF MEDICAL SCIENCE.

### PATHOLOGY AND PRACTICE OF MEDICINE.

*Oil of Turpentine in the Treatment of Yellow Fever.*—Mr. JAMES LAIRD, Surgeon R. N., who was for nearly eleven years in constant service on the North American and West Indian Station, and for several years attached to the Royal Naval Hospital, at Bermuda, bears strong testimony, (in the London Lancet, 27th August, 1853,) to the success of the turpentine treatment in the yellow fever, as it prevailed at Bermuda. Attention was first directed to this remedy, with a view to restrain passive hæmorrhage, when its salutary influence was so strikingly observed, that "it was afterwards given in every case and in every stage of the disease," and with a marked improvement in the mortality. The dose was twenty minims in camphorated water, three times a day; "and in the generality of cases nothing else was given." The following statistics tell favorably for the success of this treatment:

"*Statistics of Mortality.*

1. Showing the comparative success of different modes of treatment in 164 cases of fever, as they are entered in the books of the Royal Naval Hospital, Bermuda, from the commencement of the epidemic; and the same number of cases, taken in the same order from the 27th of August, 1843, when the spirit of turpentine was first administered:—

| *Treatment without Turpentine.* | | | *Treatment with Turpentine.* | | |
|---|---|---|---|---|---|
| No. of cases. | Died. | Rates of mortality. | No. of cases. | Died. | Rates of mortality. |
| 164 | 25 | 1 in 6·6 | 164 | 19 | 1 in 8·6 |
| Recoveries from black vomit 1. | | | Recoveries from black vomit 4. | | |

2. Showing the number of cases treated with turpentine at the Royal Naval Hospital, Bermuda, between the 27th of August and the cessation of the epidemic in December, 1843:—

| Total number of cases. | Died. | Rates of mortality. |
|---|---|---|
| 882 | 80 | 1 in 11. |
| Recoveries from black vomit . . | | 24. |

3. Showing the number of cases of yellow fever admitted into the

Royal Naval Hospital, Bermuda, during the epidemics which prevailed there in 1818, 1819, and 1837 :—

| | No. of cases. | Died. | Rates of mortality. |
|---|---|---|---|
| 1818 .. | 105 .. | 28 .. | 1 in 3·8. |
| 1819 .. | 106 .. | 25 .. | 1 in 4·2. |
| 1837 .. | 140 .. | 22 .. | 1 in 6·4." |

[The oil of turpentine is an old and highly esteemed remedy in Philadelphia practice in the treatment of yellow fever. Dr. Rush, in his history of the yellow fever which prevailed in this city in 1805, says that Dr. Physick used it that year with success for the vomiting that occurs in the second stage. Drs. Chapman and Hewson employed it at the Bush-hill hospital, in the epidemic of 1820, with great success, having cured twelve out of sixteen patients with drachm doses of the oil every hour. Prof. Samuel Jackson, in his account of the epidemic of 1820, (Philadelphia Journal of the Medical and Physical Sciences, vol. II,) speaks favorably of the effects of the oil of turpentine, although not disposed to assign it a particular pre-eminence over other remedies. He says, that "in the Hospital there were thirteen cases of the fever, in which the exhibition of turpentine was early commenced, and in which a fair trial of the practice was given. Of those cases, eight recovered and five died. (Op. citat. pp. 14, 15)." Our readers will find some interesting remarks on the use of oil of turpentine in yellow, remittent, and typhus fevers, and in diseases of a hæmorrhagic character, by Dr. S. Jackson, (late of Northumberland,) in the April number of this Journal for 1850.—Eds. Ex.]

